# Multicausal disruption of complement system activity in schizophrenia: abnormal transcription of C4, complement control proteins and microglia specific genes in brain and blood

**DOI:** 10.1192/j.eurpsy.2024.146

**Published:** 2024-08-27

**Authors:** R. Rey, A. Fiorito, E. C. Ibrahim, E. Fakra, G. Sescousse, R. Tamouza, M. Leboyer

**Affiliations:** ^1^Schizophrenia Expert Center of Lyon, CH Le Vinatier; ^2^PSYR2 team, Centre de Recherche en Neurosciences de Lyon, Bron; ^3^Institut de neurosciences de la Timone, Marseille; ^4^Fondation FondaMental, Créteil, France

## Abstract

**Introduction:**

The *synaptic pruning* process is based on the joint action of the complement system and microglia. In schizophrenia, accumulating evidence support that abnormal synaptic pruning during adolescence may be due to an altered Complement system activity. While this hypothesis is supported by *C4* overexpression in various brain regions of individuals with schizophrenia, such alterations should be replicated and extended to other brain regions. Moreover, transcriptional studies of genes encoding regulators of the complement system activity (complement control proteins, CCP) and microglia-specific genes are lacking. Furthermore, it remains unknown whether brain and peripheral expression of such genes are related.

**Objectives:**

To explore expression of *C4* as well as 4 CCP encoding genes and 10 microglia-specific genes at the brain and peripheral levels in individuals with schizophrenia as compared to healthy controls.

**Methods:**

We analyzed candidate gene expression from 9 Gene Expression Omnibus datasets obtained from 333 individuals with schizophrenia and 306 healthy controls (HC). We first compared expression of the candidate genes between individuals with schizophrenia and HC in postmortem brain samples from 7 different brain regions. Then, the same comparison was made in 4 different peripheral tissues.

**Results:**

Regarding the complement system, we observed *C4* overexpression in the DLPFC, parietal, temporal cortex and associative striatum of individuals with schizophrenia. We report distinct altered expression patterns of CCP genes in the DLPFC, hippocampus and cerebellum of individuals with schizophrenia. Only *CD46* expression was altered in the blood of individuals with schizophrenia. Regarding microglia, we report an underexpression of several microglia-specific genes in the cerebellum, associative striatum, hippocampus and parietal cortex of individuals with schizophrenia vs. HC. At the peripheral level, we observed a mixed altered expression pattern in the whole blood of individuals with schizophrenia.

**Conclusions:**

Firstly, our results suggest that the CCP-mediated regulatory mechanisms of the Complement system are impaired in the brain of individuals with schizophrenia, potentially contributing to an excessive Complement system activity (CSA). Secondly, our results support the hypothesis of a widespread underexpression of microglia-specific genes in brain tissues of individuals with schizophrenia. Functionally, the observed transcriptional alterations may be related to the synaptic pruning impairment. Alternatively, they may translate a compensatory mechanism for neuroinflammation. In the whole blood, the altered transcriptional pattern may represent a potential peripheral signature of SZ.

**Disclosure of Interest:**

None Declared

